# Enhanced Efficacy of Sandblasted, Large-Grit, Alkaline-Etched (SLA) Titanium Surfaces Combined with Plasma Treatment and Secretory Leukocyte Protease Inhibitor (SLPI) Coating in Promoting Osteoblast Activity and Reducing Bacterial Adhesion

**DOI:** 10.3390/jfb17090455

**Published:** 2026-09-07

**Authors:** Wannapat Chouyratchakarn, Nattikan Thongjui, Chayanisa Phutiyothin, Onnicha Srisopar, Surasak Tangkamonsri, Chakrit Wiboonsuntharangkoon, Pakorn Sang-Ngam, Norrapon Vichiansan, Nithi Atthi, Chayarop Supanchart, Sarawut Kumphune

**Affiliations:** 1 Biomedical Engineering Institute, Chiang Mai University, Chiang Mai 50200, Thailand; wannapat_ch@cmu.ac.th (W.C.); nattikan.t@cmu.ac.th (N.T.); chayanisa_p@cmu.ac.th (C.P.); onnicha_sri@cmu.ac.th (O.S.); surasak_tangk@cmu.ac.th (S.T.); chakrit.w@cmu.ac.th (C.W.); pakorn.sangngam@cmu.ac.th (P.S.-N.); 2 Biomedical Engineering and Innovation Research Center, Chiang Mai University, Chiang Mai 50200, Thailand; chayarop.supanchart@cmu.ac.th; 3 Office of Research Administration, Chiang Mai University, Chiang Mai 50200, Thailand; 4 Center of integrated Engineering, Faculty of Engineering, Chiang Mai University, Chiang Mai 50200, Thailand; norrapon.v@cmu.ac.th; 5 Thai Microelectronics Center (TMEC), National Science and Technology Development Agency (NSTDA), Pathum Thani 12120, Thailand; nithi.att@nstda.com; 6 Department of Oral and Maxillofacial Surgery, Faculty of Dentistry, Chiang Mai University, Chiang Mai 50200, Thailand

**Keywords:** surface modifications, surface properties, sandblast, large-grit, and alkaline etching (SLA), plasma treatment, secretory leukocyte protease inhibitor (SLPI), osteoblasts, cell adhesion, cell differentiation, bacterial adhesion

## Abstract

The clinical success rate of titanium (Ti) implants is significantly reduced in elderly patients and by implant-associated bacterial infections. Thus, Ti surfaces were modified using sandblasted large-grit alkaline etching (SLA), argon-nonthermal plasma treatment, and recombinant human secretory leukocyte protease inhibitor (rhSLPI) coating. This study evaluated surface characteristics, human fetal osteoblast (hFOB 1.19) activities, and antibacterial efficacy. The modified surfaces exhibited increased roughness and hydrophilicity compared to Ti. The SBPTi-rhSLPI enhanced 7-day protein retention on the surface. While Ti promoted higher cell proliferation, the modified groups demonstrated superior cell adhesion and mineralization. Specifically, SBPTi-rhSLPI showed the highest cytoplasmic spreading, adhesion-related gene expression (*Itg*α*1*, *Itg*α*2*, *Itg*α*5*, and *Itg*β*1*), and mineralization. Additionally, rhSLPI-coated groups effectively reduced the adhesion of both *S. aureus* and *E. coli*. In conclusion, combining SLA modification, argon plasma treatment, and rhSLPI coating provides enhanced effects. This combined approach significantly enhances osteoblast adhesion and mineralization while possessing antibacterial potential, which could be a promising strategy to improve implant success rates.

## 1. Introduction

Titanium and its alloys are widely used in orthopedic and dental implants due to their properties, which are chemical stability, excellent strength, low immunogenicity, and biocompatibility [1,2]. While the success rate of titanium (Ti) implants exceeds approximately 90% in healthy patients, this rate is significantly reduced under compromised clinical conditions [3]. However, the general properties of titanium may not be sufficient for successful implantation as the world has entered an aging society, where the elderly exhibit decreased bone density and decreased healing efficacy [4]. Additionally, the surface characteristics of implants, including surface charge, morphology, chemistry, structure, hydrocarbon contamination, and wettability, are essential for the success of the implantation [5,6]. Currently, various strategies exist to enhance the success rate of implantation, with surface modification being one of the most well-known approaches [6]. A variety of surface modifications have been developed to improve osseointegration, which can be mainly categorized into physical methods such as sandblasting and laser ablation, chemical methods such as acid/alkaline etching and anodization, and biological coatings such as hydroxyapatite and bioactive proteins [7]. The titanium surface modification has been used to improve surface properties, including improving surface roughness, hydrophilicity, and microorganism decontamination, etc., to be more suitable for enhancing osseointegration and stability after implantation [8].

One of the well-known techniques for titanium surface modification is the sandblast, large-grit, and alkaline etching (SLA), which has been used to improve the micro- and submicrometer three-dimensional structure on the surface using Al_2_O_3_ particles and etching with a basic solution [9]. Moreover, a previous study revealed that SLA could enhance the physical and chemical properties, including increasing surface roughness, hydrophilicity, and surface energy, resulting in improved cell adhesion, proliferation, and bone differentiation, which could increase osseointegration. The combination of sandblasting with large-grit and alkaline or acid etching (SLA) has also been well studied. Moreover, the previous study revealed that SLA-treated titanium surfaces increase early-stage bone healing by enhancing bone-implant contact (BIC) development and improving implant stability [10]. Furthermore, plasma treatment is another widely known strategy for titanium surface modification.

Plasma is defined by its ionized gaseous form, consisting of diverse reactive entities [11,12]. Plasma technology has been widely used for surface treatments to achieve hydrocarbon decontamination, enhance surface energy, improve wettability, and sterilize surfaces [13,14]. Argon-based non-thermal plasma treatment has been used to treat the titanium surface to enhance adhesion, proliferation, and mineralization of the osteoblast and periodontal ligament fibroblast-like cells [15]. Moreover, the titanium was treated with plasma-enhanced chemical vapor deposition (PECVD), using argon-based non-thermal plasma in a vacuum, which could enhance osteoblast adhesion [16]. Therefore, in this current study, sandblasting, large-grit alkaline etching (SLA), and argon-based non-thermal plasma treatment have been used to modify the titanium surface. However, the reduction in success rates of implantation often observed in elderly patients is another adverse condition that can further compromise the outcome. Post-implant inflammation is an inflammatory response to bacterial infections that occur after implantation [17].

Previous studies revealed that secretory leukocyte protease inhibitor (SLPI) provides antibacterial effects [18]. In addition, SLPI also has a remarkable effect on reducing cell death from apoptosis, as well as on antifungal and antiviral infections, and on improving wound healing [18,19,20]. Furthermore, the SLPI has a significant effect on the dental and orthopedic implantation. The previous studies revealed that the SLPI-coated titanium enhances bone cell adhesion, proliferation, mineralization, and differentiation [21,22]. Furthermore, applying an rhSLPI coating to titanium screws has been shown to enhance osseointegration in in vivo model [23]. However, the previous studies investigated the osteogenic effect on non-modified smooth titanium surfaces [21] or used an advanced chemical approach involving a plasma-polymerized interlayer for covalent coupling on alkaline-etched titanium [22]. Therefore, the present study investigated a combined surface modification, namely SLA and plasma treatment. SLPI has been proposed as a potential coating agent due to its diverse biological effects. While physical and chemical modifications can improve initial cell attachment, they often lack active defense mechanisms against bacterial colonization. In contrast, standalone biological coatings frequently exhibit low surface retention. Therefore, an enhanced combination of physical (SLA), chemical (PECVD), and biological (rhSLPI) modification was preferred in this current study to overcome the limitations of single-method approaches. In this combined strategy, SLA provides an optimal topography mimicking the natural bone structure, plasma increases surface energy and protein retention, and rhSLPI provides antibacterial defense and improves osteoblast activities. Thus, the aim of the present study was to determine the combined effects of SLA-modified titanium combined with argon non-thermal plasma treatment and recombinant human SLPI (rhSLPI) coating on enhancing human osteoblast activities, including adhesion, spreading, proliferation, and differentiation and antibacterial adhesion efficacy. Accordingly, the null hypotheses of this study were that these combined surface modifications would not significantly affect human osteoblast activities and would not reduce bacterial adhesion compared to the unmodified titanium surface.

## 2. Materials and Methods

**Figure 1:**
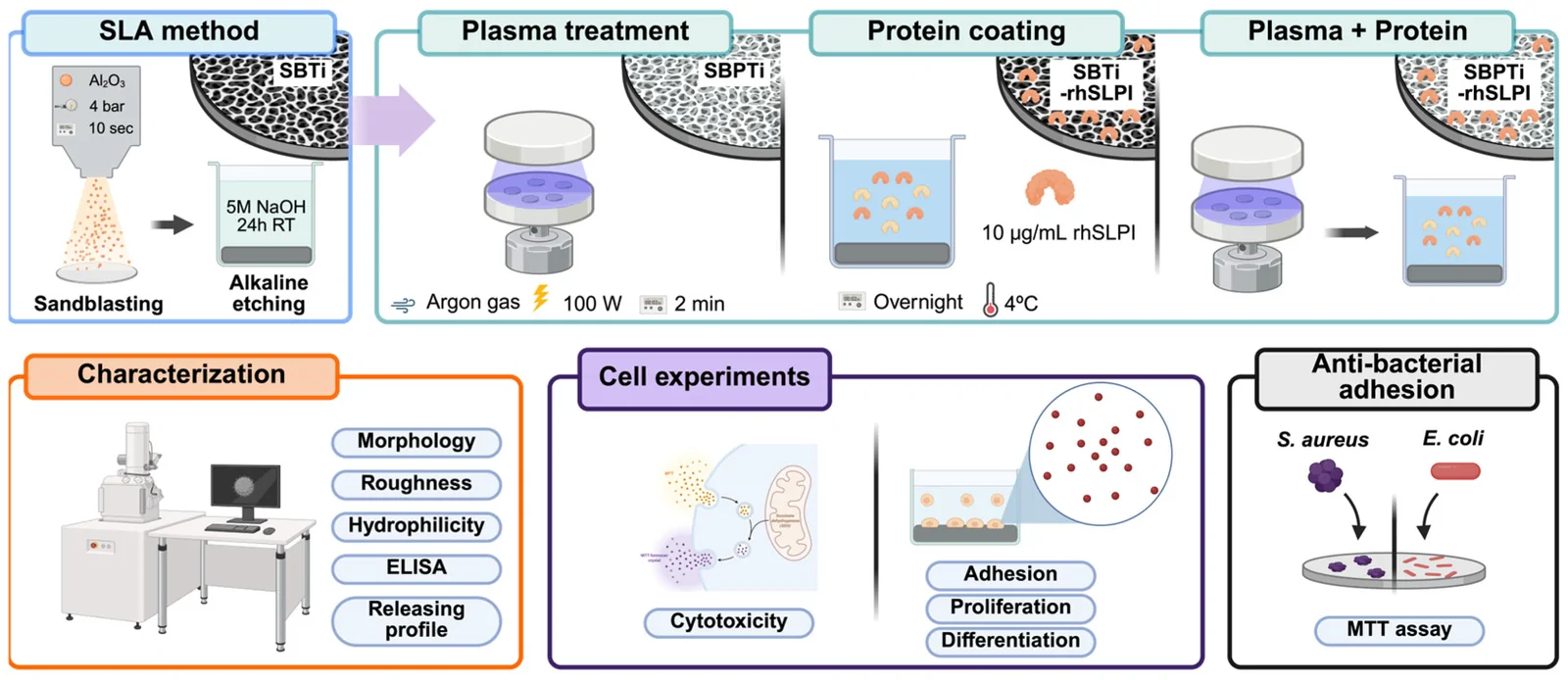
Schematic illustration of the experimental design. Titanium was modified by SLA modification (SBTi), followed by argon non-thermal plasma treatment (SBPTi), rhSLPI protein coating (SBTi-rhSLPI), or a combination of plasma treatment and rhSLPI coating (SBPTi-rhSLPI). The characteristics determined were surface morphology, roughness, hydrophilicity, protein adsorption, and release profile. Cell experiments were performed to determine cytotoxicity, adhesion, proliferation, and mineral deposition on human osteoblast cells. The effect of antibacterial adhesion was determined against Gram-positive and Gram-negative bacteria.

The experimental design of this study was mainly divided into four parts: the fabrication step, surface characteristics, effect on human osteoblast cells, and antibacterial adhesion (Figure 1).

### 2.1. Chemicals and Reagents

Recombinant human secretory leukocyte protease inhibitor (rhSLPI) was purchased from Sino Biology Inc. (Beijing, China), and Dulbecco’s modified Eagle’s medium (DMEM): Ham’s F-12 medium, fetal bovine serum (FBS), and trypsin-EDTA were purchased from Gibco BRL; Life Technologies Inc. (New York, NY, USA).

### 2.2. Cell Culture and Subculture

The human fetal osteoblast 1.19 (hFOB 1.19) cells were purchased from American Type Cell Culture (ATCC CRL-11372^TM^). Cells were cultured in a 1:1 mixture of Ham’s F12 Medium and Dulbecco’s Modified Eagle’s Medium, containing 2.5 mM L-glutamine (without phenol red), supplemented with 10% (*v*/*v*) fetal bovine serum (FBS), 1% of 5000 units/mL penicillin, and 5000 µg/mL streptomycin (Gibco^®^). For culture conditions, the hFOB 1.19 cells were cultured at 37 °C with 5% carbon dioxide (CO_2_) and 95% air.

### 2.3. Sample Preparation

Grade 5 titanium disks (Ti) were machined to 5.5 mm in diameter and 0.5 mm in thickness, and 12 mm in diameter and 0.5 mm in thickness for the 96-well plate and 24-well plate, respectively. The Ti was washed three times with acetone, ethanol, and ultrapure water (10 min in each step) in an ultrasonic bath. Then the Ti was sterilized by autoclave and placed in a hot air oven until dry.

The Ti disks were separated into five groups:

- Non-modified Ti (Ti);
- Sandblasted, large-grit, and alkaline etching (SLA) Ti (SBTi);
- The combination of SLA and plasma treatment on Ti (SBPTi);
- The combination of SLA and rhSLPI coating (SBTi-rhSLPI);
- The combination of SLA, plasma treatment, and rhSLPI coating (SBPTi-rhSLPI).

The SBTi samples were prepared using the sandblast, large-grit, and alkaline etching (SLA) technique, which followed from the previous study [9]. Briefly, the Ti was vertically sandblasted with 120 µm Al_2_O_3_ particles at a pressure of 4 bar for 10 s at a distance of 10 cm from the surface. Then the sandblasted Ti was applied to a 5M NaOH solution for 24 h at room temperature. After the incubation, the SBTi were washed with ultrapure water for 10 min in an ultrasonic bath thrice.

The SBPTi samples were prepared by the combination of SLA and plasma treatment. Briefly, the Ti disks were first modified by SLA under the same conditions as above. Subsequently, the SBTi was treated with argon plasma under vacuum (pressure of 1.1 × 10^−1^ Torr) for 2 min at 100 W. Then the plasma-treated SBTi (SBPTi) was transferred to a sterile container.

The SBTi-rhSLPI were prepared by coating the rhSLPI protein on the SBTi samples. For rhSLPI immobilization, the surface-modified samples were coated with 10 µg/mL of rhSLPI in phosphate-buffered saline (PBS), which was shown to be the optimal concentration for improving human osteoblast cell adhesion on the Ti surface [21]. The samples were placed into a well plate, and 10 µg/mL rhSLPI solution was then added to the well at 100 µL/well and incubated at 4 °C for 24 h.

Lastly, the combination of SLA, plasma treatment, and rhSLPI coating (SBPTi-rhSLPI) was performed in the same way as the above procedure.

### 2.4. Surface Characterization

The physical characteristics of Ti were observed by three readout measurements: surface morphology, surface roughness, and surface hydrophilicity of the Ti surface.

The surface morphology was observed by a field emission scanning electron microscope (FE-SEM, JSM-IT800; JEOL, Tokyo, Japan). Briefly, the Ti samples were placed on a stub and then coated with gold for 30 s.

The surface roughness of the Ti surface was measured using a 3D optical profilometer (Contour X-500; Bruker, MA, USA). The roughness value was calculated as average roughness (Pa) and compared to control groups.

The hydrophilicity of the Ti surfaces was determined using the sessile drop assay. First, 0.5 µL of deionized water was dropped on the Ti surface at 25 °C, and then the angle of the water droplet was measured using a drop shape analysis system (Model SL150, Kino, Tokyo, Japan) with CAST 3.0 software. The measurement range was 0–180° with a 0.01° resolution, and the contact angles of completely spreading droplets (superhydrophilic) were recorded as 0° for statistical analysis. The water contact angle of test groups was analyzed and compared to control groups.

### 2.5. Protein Immobilization Assay

After rhSLPI coating, the confirmation of protein adsorption on the Ti surface was determined using a single-wash sandwich enzyme-linked immunosorbent assay (ELISA) kit (Abcam, Cambridge, UK). The process was modified from the manufacturer’s instructions. Briefly, the Ti was coated with 10 µg/mL rhSLPI for 24 h at 4 °C. Then the rhSLPI-coated Ti was washed with PBS three times for 5 min each and transferred into a new 96-well plate. The rhSLPI was captured by an anti-rhSLPI antibody conjugated with horseradish peroxidase (HRP) for 1 h at room temperature on a shaker. After the incubation period, the coated Ti was washed three times with 350 µL of washing buffer. Then the 100 µL tetramethylbenzidine (TMB) working solution was added and incubated for 10 min in the dark. Lastly, the 100 µL stop solution was added, and the absorbance was determined at O.D. 450 nm by a microplate spectrophotometer (Infinite M Plex; TECAN, Männedorf, Switzerland). The absorbance value was analyzed and compared to control groups.

### 2.6. The rhSLPI Release Profile

The release of rhSLPI was evaluated by incubating the Ti samples in phosphate-buffered saline (PBS) for 7 days. At predetermined time points (days 0, 1, 3, 5, and 7), a 200 µL aliquot of the solution was collected and immediately replaced with an equal volume of fresh PBS. The rhSLPI concentration was quantified using an ELISA kit (Abcam, Cambridge, UK). The results were expressed as the cumulative relative percentage of release, which was calculated relative to the initially adsorbed amount of the rhSLPI on the surface.

### 2.7. Cytotoxicity Assay

The cytotoxicity of samples on osteoblasts was evaluated using the MTT cell viability assay. Firstly, the samples were placed into a 96-well plate. Then, 200 µL of 5 × 10^5^ cells/mL hFOB 1.19 cells were seeded on the Ti surface and incubated at 37 °C with 5% CO_2_ and 95% air for 24 h. After the incubation, the culture medium was removed, and 0.5 mg/mL MTT solution was added and incubated for 2 h at 37 °C with 5% CO_2_ and 95% air. Then the 0.5 mg/mL MTT solution was discarded, and DMSO solution was applied to dissolve the formazan crystal. Finally, the absorbance was determined by spectrophotometer (O.D. 570 nm), and the O.D. value was calculated to the relative percent of cell viability.

### 2.8. Cell Adhesion Assay

The adhesion of osteoblast cells was determined using the MTT assay. A total of 200 µL of 2.5 × 10^5^ cells/mL hFOB 1.19 cells was seeded on Ti disks and incubated at 37 °C with 5% CO_2_ and 95% air for 20 min, which was optimized for optimal hFOB 1.19 cell adhesion [21,24]. After the incubation period, the culture medium was replaced with new fresh medium and continuously incubated for 1 h. Then, the culture medium was discarded, and 0.5 mg/mL MTT solution was added and incubated for 2 h at 37 °C with 5% CO_2_ and 95% air. The MTT solution was discarded, and the formazan crystals were dissolved using DMSO and determined the absorbance (O.D. 570 nm) by spectrophotometer. The O.D. value was directly proportional to the number of cells adhered on the surface.

### 2.9. Cell Morphology Observation

The hFOB 1.19 cells were trypsinized and seeded onto the 12 mm-diameter Ti disks in a 24-well plate at 1 × 10^5^ cells/mL. The cells were then incubated at 37 °C with 5% CO_2_ and 95% air for 20 min. After the incubation, the culture medium was removed and the cells were washed with PBS three times. The cells were fixed with 2.5% (*v*/*v*) glutaraldehyde for 30 min at room temperature. Subsequently, the cells were dehydrated with gradients of ethanol (50, 70, 90, and 100% (*v*/*v*)) for 10 min per concentration. Then, the Ti disks were transferred to a new 24-well plate and dried overnight. Finally, the morphology was observed under an FE-SEM.

### 2.10. Gene Expression

The gene expression of the adhesion molecule was analyzed using quantitative reverse transcription polymerase chain reaction (qRT-PCR). Firstly, the hFOB 1.19 cells were seeded on Ti disks at a concentration of 1.5 × 10^4^ cells/mL and incubated for 7 days at 37 °C with 5% CO_2_ and 95% air. After the incubation, the cells were harvested and RNA was extracted using a commercial RNA extraction kit (PureLink^®^ RNA Mini Kit, Ambion, Austin, TX, USA). The concentration and purity of RNA were measured by a spectrophotometer (Nanodrop, Thermo, Waltham, MA, USA) at wavelengths of 260 nm and 280 nm. The mRNA was converted to cDNA using a Viva cDNA Synthesis Kit (Vivantis, Selangor Darul Ehsan, Malaysia). The expression of *Itgα1*, *Itgα2*, *Itgα5*, and *Itgβ1* gene was determined using a SensiFAST^™^ SYBR^®^ No-ROX kit (Bioline, London, UK) and the primer list in Table 1. The PCR results will be normalized to GAPDH, which is an internal control and housekeeping gene, based on the 2^−ΔΔCt^ [25].

### 2.11. Cell Proliferation

The proliferation assay was performed using the Bromodeoxyuridine (BrdU) incorporation assay. The process was conducted following the manufacturer’s instructions. Briefly, the hFOB 1.19 cells were seeded onto Ti disks at a concentration of 2.5 × 10^4^ cells/mL and incubated at 37 °C with 5% CO_2_ and 95% air for 3 and 5 days. On day 3 and day 5, the BrdU reagent was added to the cells and incubated for 2 h. Then, the culture medium was discarded, and the cells were fixed with a fix solution. Subsequently, anti-BrdU monoclonal detector antibody was added and incubated for 1 h at room temperature. After incubation, the solution was discarded, and the peroxidase goat anti-mouse IgG conjugate was added to the cells. After 30 min of incubation, the TMB peroxidase substrate was added and incubated for 30 min. Then, the reaction was stopped, and the absorbance (O.D. 450/550 nm) was determined by a spectrophotometer.

### 2.12. Osteogenic Differentiation Assay

Osteoblastic differentiation was performed using osteogenic differentiation medium. Firstly, the 12 mm-diameter Ti disks were placed into a 24-well plate, and 1 × 10^5^ cells/mL hFOB 1.19 cells were seeded on the Ti disks. The cells were incubated for 24 h at 37 °C with 5% CO_2_ and 95% air. Then the culture medium was discarded, and the osteogenic differentiation medium (100 nM dexamethasone, 10 mM sodium β-glycerophosphate, and 100 µg/mL ascorbic acid) was added. Moreover, the osteogenic differentiation medium was renewed every three days. The cells were differentiated for 7 days for osteogenic gene expression and 21 days for mineralization.

### 2.13. Mineralization Assay

After the osteoblast cells were differentiated for 21 days, the cells were fixed with 4% (*v*/*v*) paraformaldehyde at room temperature for 15 min and washed three times with PBS. Then the cells were stained with 2% (*w*/*v*) alizarin red S for 20 min at room temperature. After staining, the staining solution was discarded, and the Ti was washed with PBS and air-dried. Finally, the staining was solubilized with 10% (*w*/*v*) cetylpyridinium chloride monohydrate solution, and the absorbance (O.D. 570 nm) was measured using a microplate spectrophotometer.

### 2.14. Antibacterial Adhesion Assay

The antibacterial adhesion efficacy against *Staphylococcus aureus* (*S. aureus*) and *Escherichia coli* (*E. coli*), which serve as representative Gram-positive and Gram-negative models, was evaluated using the MTT assay. Initially, the bacterial strains were cultured in Luria–Bertani (LB) broth at 37 °C in a shaking incubator until the optical density at 600 nm (OD600) reached 0.1. Subsequently, the specimens were placed into a 24-well plate, inoculated with the bacterial suspension, and incubated at 37 °C for 24 h. Following incubation, the samples were transferred to fresh sterile tubes containing 5 mL of PBS. To detach the adhered bacteria from the surfaces, the samples were sonicated for 15 min. The resulting suspension was then centrifuged at 3500 rpm for 15 min. Following centrifugation, the bacterial pellet was resuspended in 500 µL of PBS. Subsequently, 200 µL of the bacterial suspension was transferred to a 96-well plate for the MTT assay. The relative viability of the adhered bacteria was quantified by measuring the absorbance at 570 nm.

### 2.15. Statistical Analysis

Statistical analyses were performed using GraphPad Prism software (version 10; GraphPad Software, San Diego, CA, USA). All data are presented as the mean ± standard deviation (SD). Any data points falling outside the range of the mean ± 2 standard deviations (SD) were identified as statistical outliers and subsequently excluded from the analytical process. The normal distribution of the data was verified prior to analysis. Statistical significance between groups was evaluated using a one-way or two-way analysis of variance (ANOVA), followed by the Tukey–Kramer test for multiple comparisons. A *p*-value < 0.05 was considered statistically significant.

## 3. Results

### 3.1. Surface Characteristics

**Figure 2:**
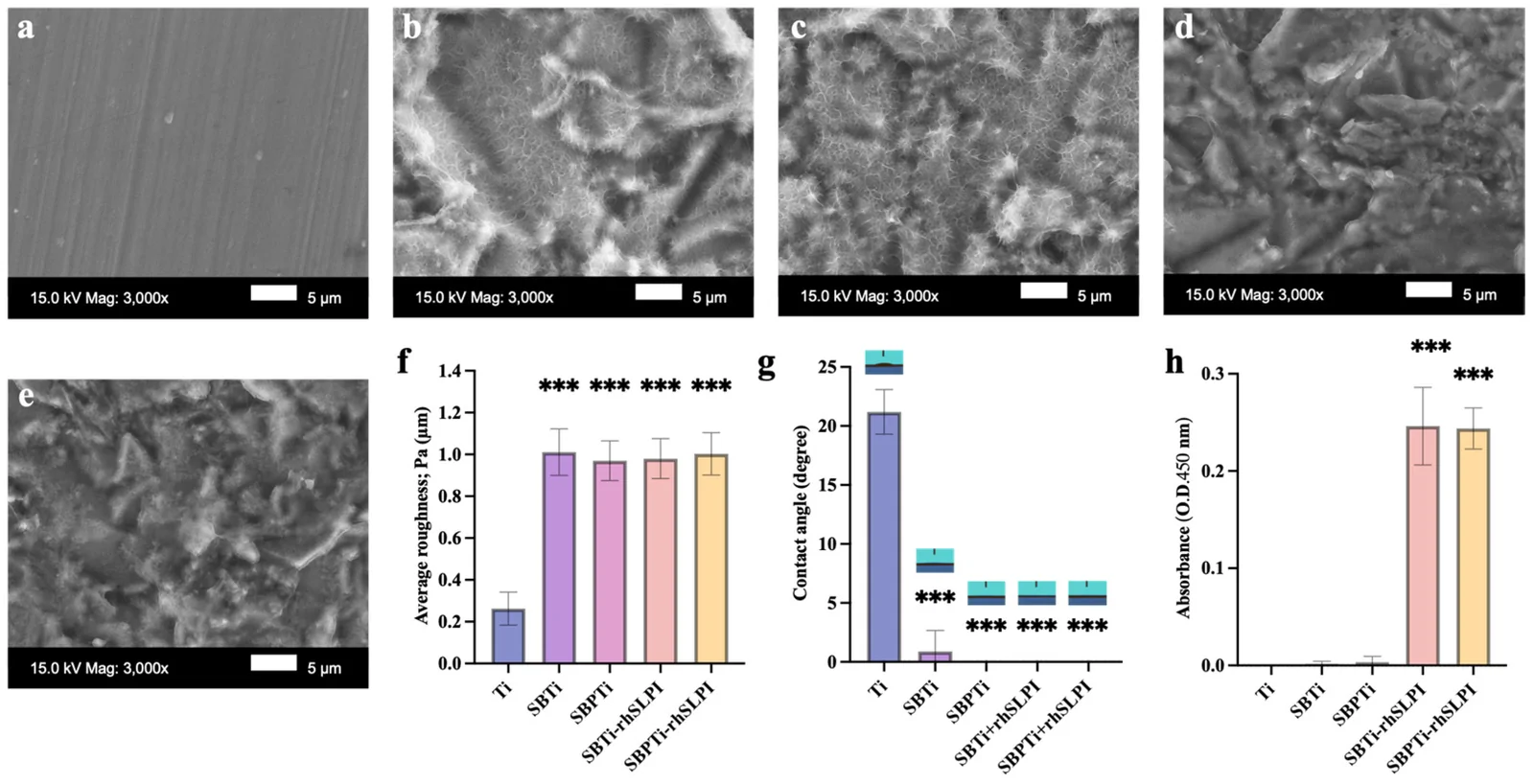
The surface characteristics. The surface morphology of Ti disks was observed under a scanning electron microscope (SEM), including (**a**) Ti, (**b**) SBTi, (**c**) SBPTi, (**d**) SBTi-rhSLPI, and (**e**) SBPTi-rhSLPI. (**f**) The average roughness of the Ti surface was observed under a 3D optical profilometer. (**g**) The sessile drop assay revealed the hydrophilicity of the Ti surface. (**h**) The ELISA was performed to confirm the rhSLPI on the surface. *** *p* < 0.001 vs. control (One-way ANOVA) (*n* = 6 per group).

The surface characteristics of the Ti disks, including surface morphology, surface roughness, hydrophilicity, and ELISA, were evaluated (Figure 2). After the preparation, the morphology of Ti was observed under a field emission scanning electron microscope (FE-SEM). The surfaces of Ti (control) showed smooth surfaces with minor scratches (Figure 2a). In contrast, the SEM revealed that the surfaces of the Ti disk after the SLA modification, including SBTi, SBPTi, SBTi-rhSLPI, and SBPTi-rhSLPI, showed a spiderweb-like surface texture (Figure 2b, c, d, and e, respectively). Then, the surface roughness was performed using a profilometer. The average roughness of modified groups, including SBTi, SBPTi, SBTi-rhSLPI, and SBPTi-rhSLPI, showed a significant increase compared to the control (Ti). However, there was no significant difference within the SLA modification groups (Figure 2f). Furthermore, hydrophilicity was measured using a sessile drop technique (Figure 2g). The contact angle of the SBTi (0.88° ± 1.77°) was significantly reduced compared to the control (Ti, 21.20° ± 1.89°). Moreover, the SBPTi, SBTi-rhSLPI, and SBPTi-rhSLPI could not be determined since the contact angle was below the limit of the measurement. The rhSLPI coating on the Ti surface was determined using ELISA (Figure 2h). The results demonstrated the absence of rhSLPI on the surface of the Ti and non-coated groups (SBTi and SBPTi), whereas rhSLPI was present on the coated groups (SBTi-rhSLPI and SBPTi-rhSLPI). However, the statistical analysis indicated that there was no significant difference compared to the SBTi-rhSLPI (0.24 ± 0.03) and SBPTi-rhSLPI (0.24 ± 0.02).

### 3.2. The rhSLPI Releasing Profile

**Figure 3:**
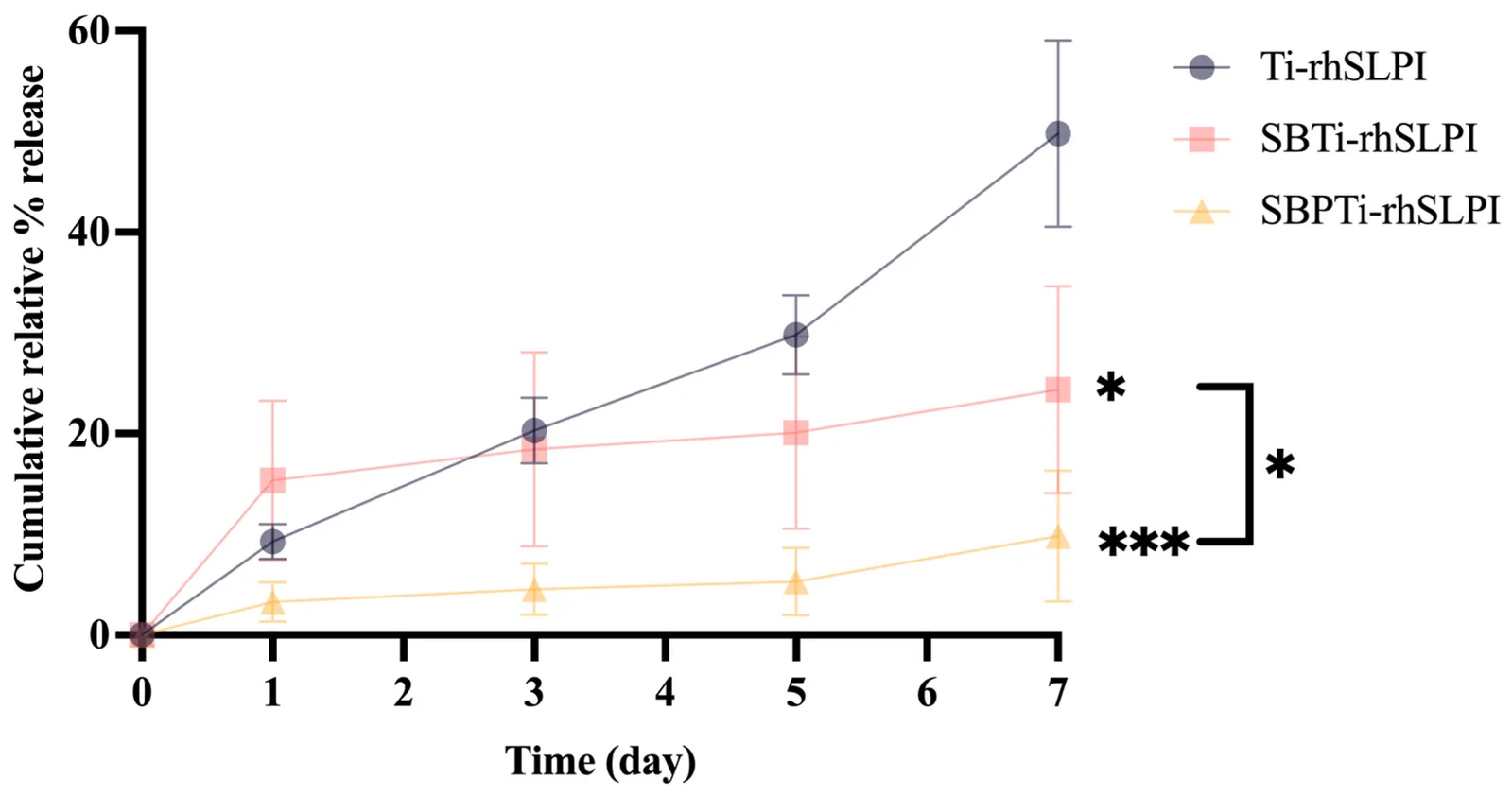
In vitro release profile of rhSLPI. The amount of released rhSLPI over a 7-day period was quantified using an enzyme-linked immunosorbent assay (ELISA). Data are presented as the cumulative relative percentage of rhSLPI release. * *p* < 0.05 vs. control and *** *p* < 0.001 vs. control (two-way ANOVA) (*n* = 3 per group).

The release profiles of the rhSLPI-coated groups were determined using ELISA over a 7-day period (at days 0, 1, 3, 5, and 7). The results demonstrated that the cumulative release from SBTi-rhSLPI (0 ± 0.00%, 15.35 ± 13.67%, 18.42 ± 16.73%, 20.10 ± 16.57%, and 24.36 ± 17.77%, respectively) and SBPTi-rhSLPI (0 ± 0.00%, 3.28 ± 1.20%, 4.52 ± 2.43%, 5.32 ± 4.78%, and 9.82 ± 7.30%, respectively) was significantly lower compared to the Ti-rhSLPI control group (0 ± 0.00%, 9.28 ± 2.98%, 20.31 ± 5.67%, 29.82 ± 6.85%, and 49.80 ± 16.01%, respectively) (Figure 3). Moreover, the results exhibited a significant difference between the SBTi-rhSLPI and SBPTi-rhSLPI groups.

### 3.3. The Cytotoxicity on Osteoblast

**Figure 4:**
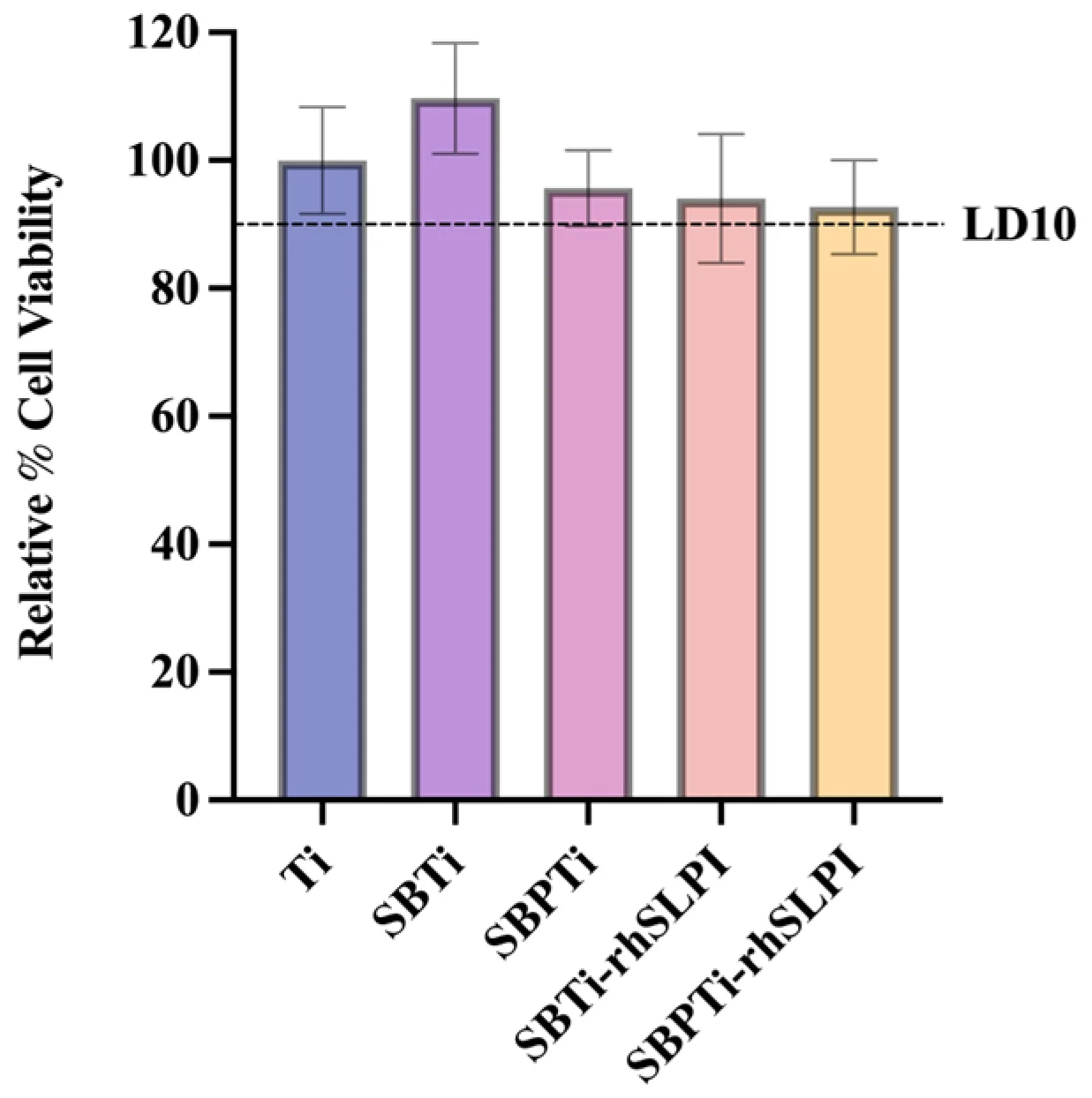
The toxicity of titanium on human osteoblast cells. The cytotoxicity of non-modified and modified titanium surfaces was determined using an MTT cell viability assay. (*n* = 3–5 per group).

After the sample preparation, the toxicity of the titanium was determined by the MTT cell viability assay. The hFOB 1.19 cells were seeded on titanium and incubated for 24 h. The results showed that the viability of cells in the modified groups was not significantly different from that in the Ti group, which served as the control (Figure 4).

### 3.4. The Adhesion Assay

**Figure 5:**
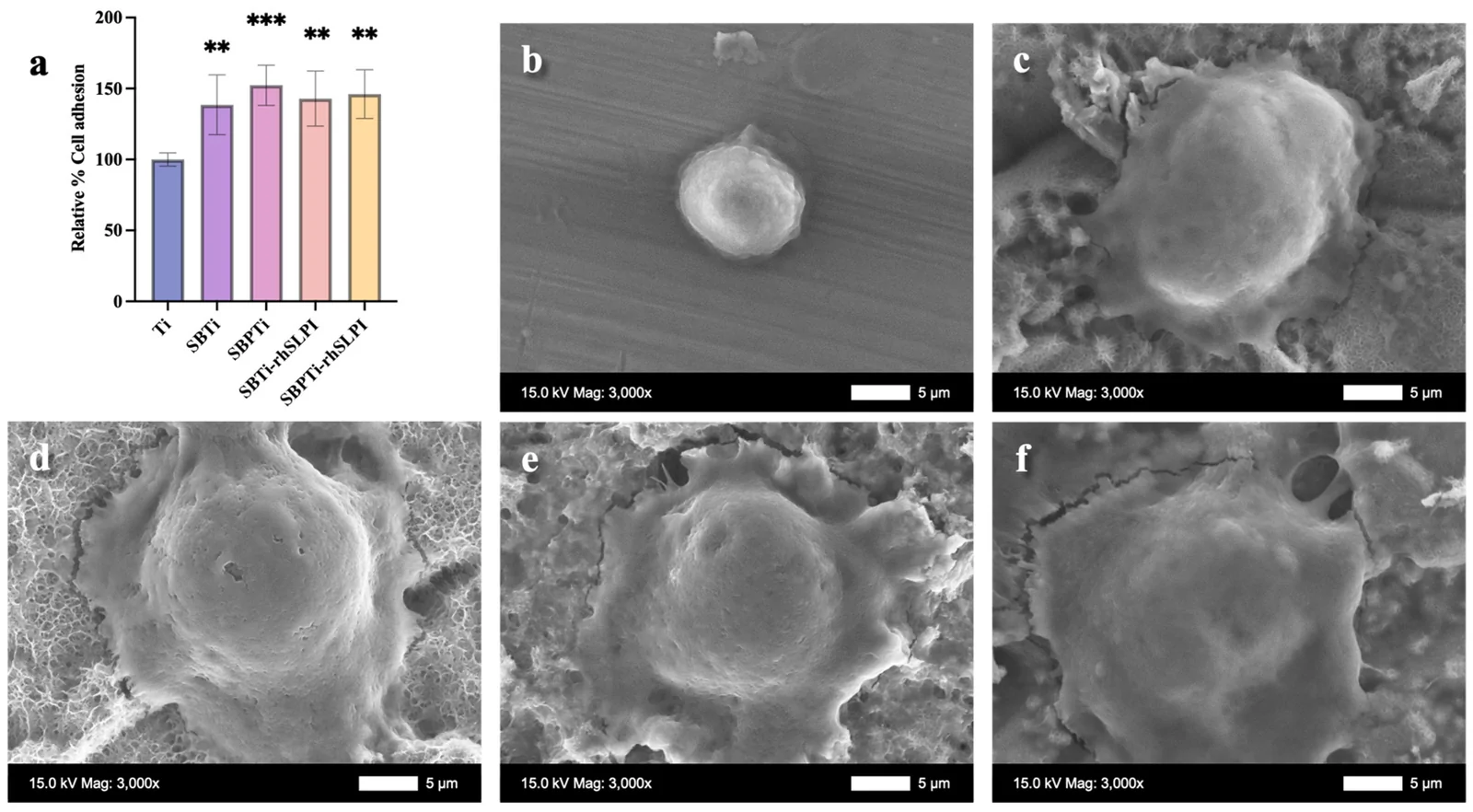
The adhesion assay. (**a**) The amount of hFOB 1.19 cell adhesion was measured by MTT assay. The morphology of osteoblast cell adhesion and spreading on the Ti surface, including (**b**) Ti, (**c**) SBTi, (**d**) SBPTi, (**e**) SBTi-rhSLPI, and (**f**) SBPTi-rhSLPI. ** *p* < 0.01 vs. control and *** *p* < 0.001 vs. control (One-way ANOVA) (*n* = 5 per group).

The effect of modified Ti on human osteoblast adhesion was determined by quantifying the number of adhered cells and observing their morphology on the Ti surface. Firstly, hFOB 1.19 cells were cultured on the Ti surface for 20 min, and the number of adhered cells was assessed using the MTT assay. The results revealed that the modified groups exhibited significantly higher cell adhesion on the Ti surface compared to the unmodified Ti control group, whereas no significant differences were observed among the modified groups (Figure 5a). The morphology of the cells after 20 min of incubation was observed under the FE-SEM. The results demonstrated that cells on the Ti surface exhibited a round morphology without cytoplasmic extension (Figure 5b). In contrast, cells on the SBTi surface showed a round morphology with initial cytoplasmic extensions (Figure 5c). Moreover, the morphology of the cells on SBPTi (Figure 5d) and SBTi-rhSLPI (Figure 5e) surfaces exhibited pronounced cytoplasmic extensions extending away from the cell body, while a round morphology was still observed in the central region of the cells. In the SBPTi-rhSLPI group, the cells exhibited pronounced cytoplasmic extensions and showed a flattened morphology (Figure 5f).

### 3.5. The Gene Expression of Adhesion Molecules

**Figure 6:**
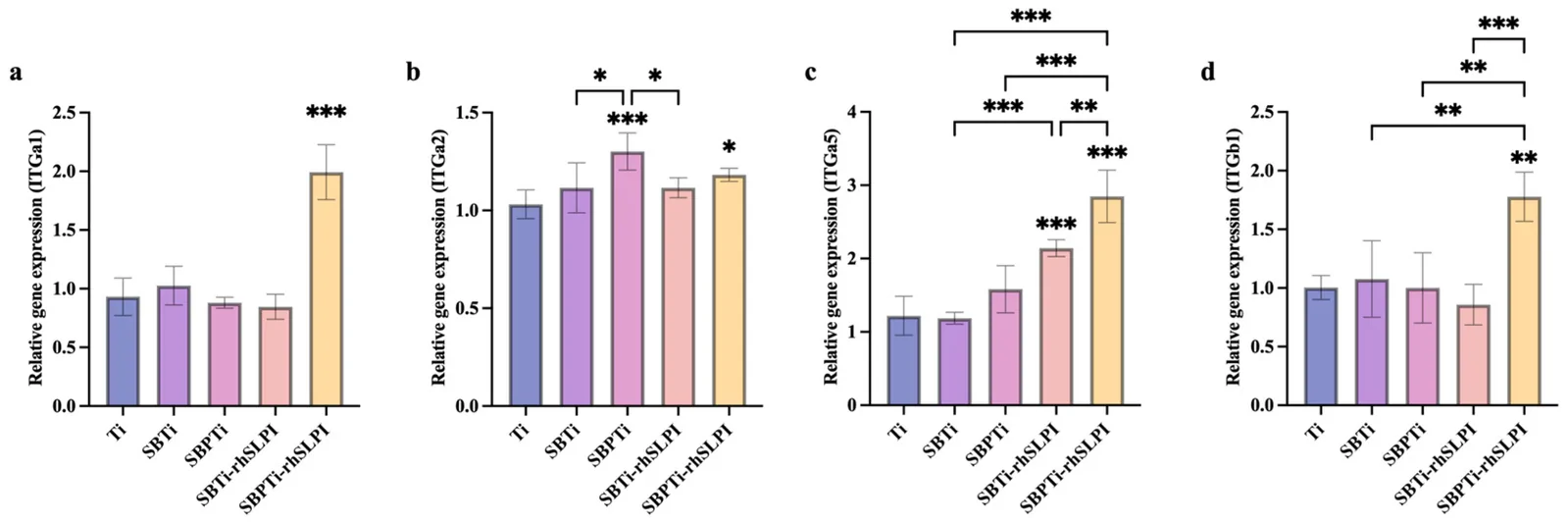
The expression of adhesion genes. The effect of surface modification on cell adhesion was revealed by examining the expression of adhesion molecules, including (**a**) *Itg*α*1*, (**b**) *Itg*α*2*, (**c**) *Itg*α*5*, and (**d**) *Itg*β*1,* using the RT-qPCR technique. * *p* < 0.05 vs. control, ** *p* < 0.01 vs. control, and *** *p* < 0.001 vs. control (One-way ANOVA) (*n* = 3–6 per group).

The expression of adhesion genes, including *Itg*α*1*, *Itg*α*2*, *Itg*α*5*, and *Itg*β*1*, was determined using RT-qPCR, and the results were presented as relative gene expression. The results showed that the *Itg*α*1* expression in the SBPTi-rhSLPI (1.99 ± 0.23) was significantly higher compared to Ti (0.93 ± 0.15) and the other modified groups (Figure 6a). Additionally, the *Itg*α*2* expression showed significantly higher levels of SBPTi (1.30 ± 0.09) and SBPTi-rhSLPI (1.18 ± 0.03) compared to the Ti control groups (1.03 ± 0.07); notably, the SBPTi exhibited greatly higher gene expression compared to SBTi (1.11 ± 0.12) and SBTi-rhSLPI (1.11 ± 0.05), whereas there was no significant difference between SBPTi and SBPTi-rhSLPI (Figure 6b). Moreover, the expression of Itgα5 on SBTi-rhSLPI (2.14 ± 0.11) and SBPTi-rhSLPI (2.84 ± 0.35) was significantly higher compared to the Ti group (1.22 ± 0.26) (Figure 6c). The cells on SBTi-rhSLPI exhibited higher expression of *Itg*α*5* compared to SBTi (1.18 ± 0.08), while SBPTi-rhSLPI demonstrated the highest *Itg*α*5* expression among the modified groups. In addition, the expression of Itgβ1 on SBPTi-rhSLPI (1.77 ± 0.21) exhibited the highest expression compared to the Ti (1.00 ± 0.10) and among the modified groups (Figure 6d).

### 3.6. The Proliferation Assay

**Figure 7:**
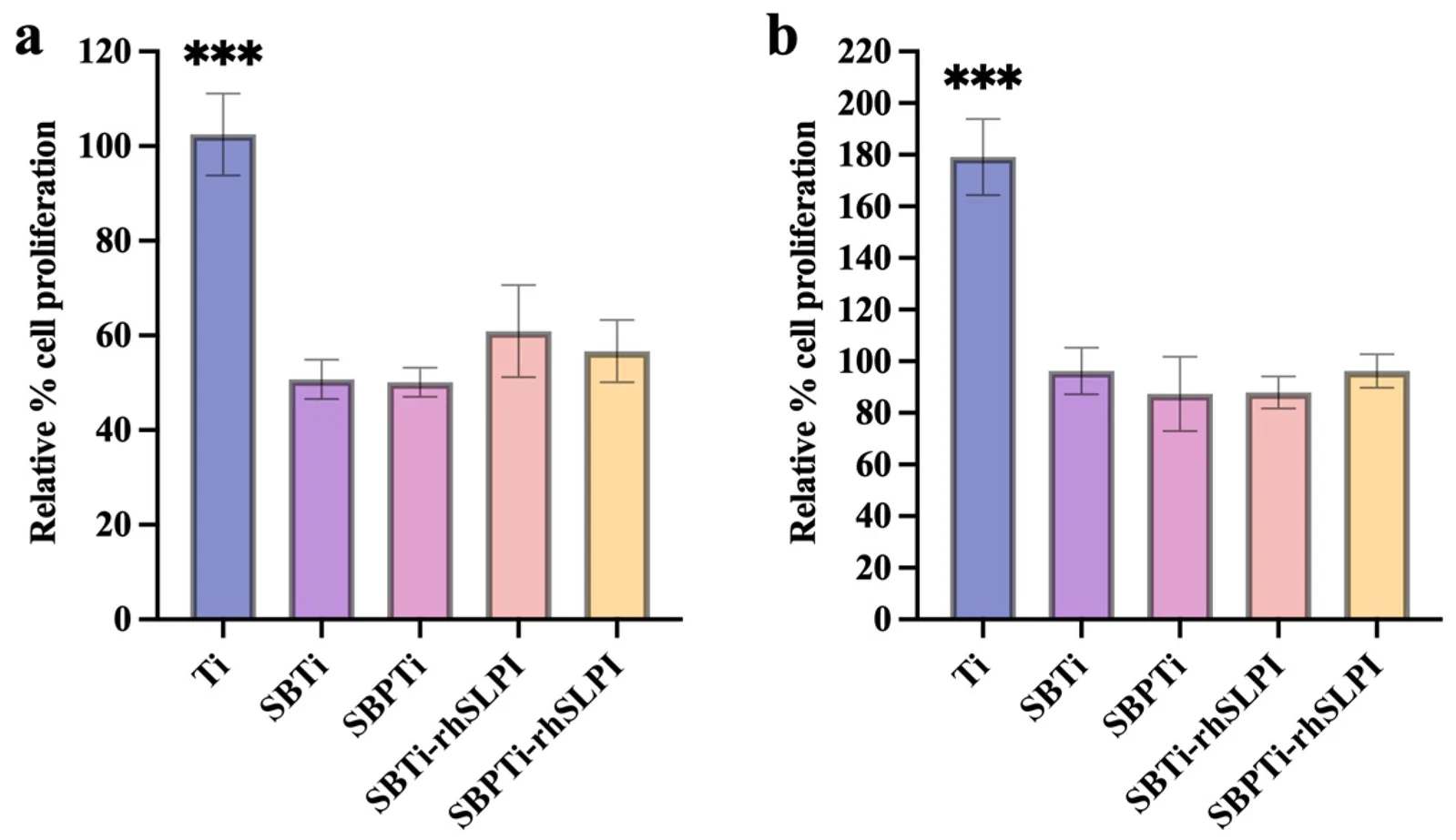
The effect of titanium on osteoblast proliferation. The BrdU assay shows the proliferation of hFOB 1.19 on (**a**) day 3 and (**b**) day 5. *** *p* < 0.001 vs. control (One-way ANOVA) (*n* = 3–6 per group).

Osteoblast cell proliferation was determined using the BrdU assay for 3 days and 5 days (Figure 7). The results showed that at day 3 and day 5 the Ti, which is the control, exhibited the highest cell proliferation compared to the modified groups, including SBTi, SBPTi, SBTi-rhSLPI, and SBPTi-rhSLPI.

### 3.7. Bone Mineralization

**Figure 8:**
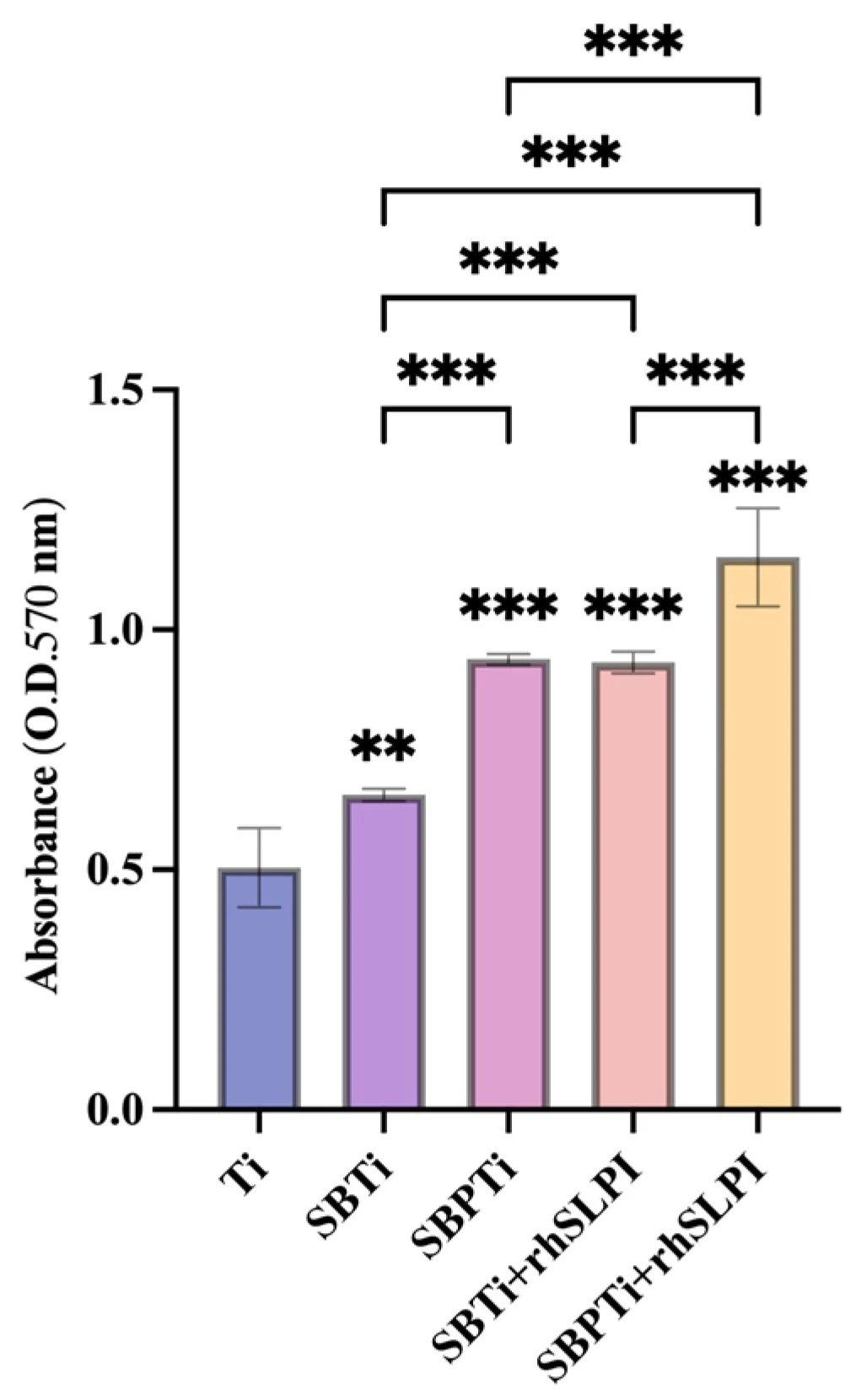
The osteoblast differentiation. The mineralization was determined by alizarin red S staining after a 21-day induction period. ** *p* < 0.01 vs. control and *** *p* < 0.001 vs. control (One-way ANOVA) (*n* = 4–6 per group).

Bone differentiation was determined by evaluating the mineralization using alizarin red S staining (Figure 8). The results showed that the modified groups, including SBTi (0.65 ± 0.01), SBPTi (0.93 ± 0.01), SBTi-rhSLPI (0.93 ± 0.02), and SBPTi-rhSLPI (1.15 ± 0.10), exhibited significantly higher deposition compared to the Ti control group (0.50 ± 0.08). Moreover, SBPTi-rhSLPI exhibited the highest mineral deposition compared to the other modified groups. Additionally, both SBPTi and SBTi-rhSLPI showed significantly higher mineral deposition compared to SBTi, with no significant difference between SBPTi and SBTi-rhSLPI.

### 3.8. Antibacterial Adhesion

**Figure 9:**
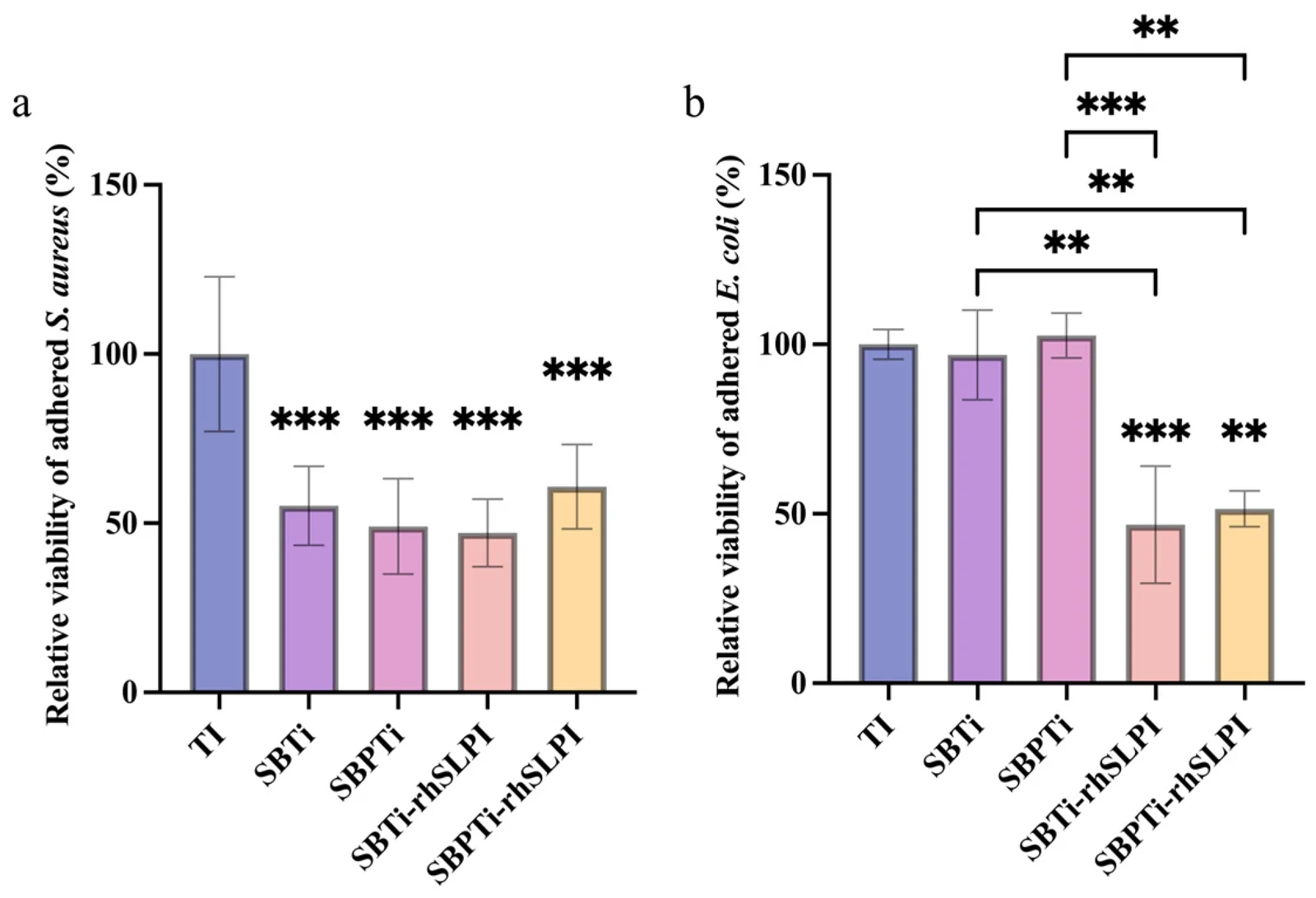
Antibacterial adhesion efficacy. The relative viability of adhered bacteria on the surface was quantified using the MTT assay. (**a**) Anti-adhesion activity against *S. aureus*. (**b**) Anti-adhesion activity against *E. coli.* ** *p* < 0.01 vs. control and *** *p* < 0.001 (one-way ANOVA) (*n* = 6 per group).

The efficacy against viable antibacterial adhesion on the modified titanium surfaces was evaluated against Gram-positive *S. aureus* and Gram-negative *E. coli* bacterial strains after 24 h of incubation using the MTT assay. The results demonstrated that all modified groups, including SBTi (55.17 ± 11.70%), SBPTi (49.06 ± 14.13%), SBTi-rhSLPI (47.16 ± 9.97%), and SBPTi-rhSLPI (60.82 ± 12.48%), significantly reduced *S. aureus* adhesion compared to the Ti control (100.00 ± 22.85%). However, there was no significant difference observed among the modified groups (Figure 9a). Moreover, for *E. coli*, the rhSLPI coating groups, including SBTi-rhSLPI (46.81 ± 17.29%) and SBPTi-rhSLPI (52.48 ± 5.31%), demonstrated a significant reduction in bacterial adhesion compared to the control (100.00 ± 4.38%), whereas SBTi and SBPTi exhibited no difference (Figure 9b).

## 4. Discussion

Implant failure in compromised clinical conditions, mainly caused by bacterial infections and insufficient bone quality in aging patients, remains a major challenge in orthopedic and dental applications. Here, the combined strategy of SLA, argon non-thermal plasma, and rhSLPI coating successfully enhances human osteoblast activities and significantly reduces bacterial adhesion on titanium surfaces.

The surface modification of Ti was observed to have the following physical characteristics: surface morphology, surface roughness, and hydrophilicity. The morphology of Ti showed a smooth surface with a few scratch lines, which occurred from the metal lathe machine during the cutting process. In contrast, the surface morphology of SLA-modified groups (SBTi, SBPTi, SBTi-rhSLPI, and SBPTi-rhSLPI) showed the spider-web-like surface texture resulting from the sandblasting and alkaline etching, and the surface texture was consistent with findings from the previous study [9]. Furthermore, the SBTi-rhSLPI and SBPTi-rhSLPI demonstrated the mucus-like film coated on the surface, which might indicate the presence of rhSLPI protein. The ELISA confirmed the presence of rhSLPI protein on SBTi-rhSLPI and SBPTi-rhSLPI surfaces, whereas no rhSLPI was detected on the surfaces of the other groups. Moreover, the surface roughness from the profilometer was consistent with surface morphology via FE-SEM. The water contact angle showed a significant decrease following SLA modification, and the previous studies suggest that this decrease involves an increase in the negative charge from the hydroxyl groups (-OH) after treatment with NaOH solution [9,26]. In addition, the plasma treatment could enhance the surface hydrophilicity via the increase in surface energy from the hydroxyl groups and reactive oxygen species (ROS) [27]. The increase in hydrophilicity after SLPI coating might be due to the positive charge and polar nature of SLPI protein, which enhances the ability of water molecules to spread on the titanium surface [18].

The release profile of rhSLPI coated on the surfaces is a crucial factor in determining the long-term efficacy of medical implants. The results demonstrated that the surface modifications significantly influenced the release kinetics of the rhSLPI protein. The control group (Ti-rhSLPI) exhibited a rapid release, reaching the highest cumulative release by day 7. This rapid release phenomenon suggests that the rhSLPI protein on the surface was immobilized through weak physical adsorption which is easily disrupted upon exposure to the physiological environment [28]. In contrast, both surface-modified groups (SBTi-rhSLPI and SBPTi-rhSLPI) demonstrated a lower cumulative release compared to the control. However, the relatively large standard deviations observed might be attributed to the irregular and micro- and nano-roughened topography generated by the SLA, which may cause non-uniform physical adsorption and sample-to-sample variability during the release phase. This provides strong evidence that the modified surface titanium effectively enhanced retention and modulated the release kinetic of the rhSLPI. Moreover, the SBPTi-rhSLPI group exhibited the most sustained and restricted release profile, with a cumulative release of under 10% throughout the entire 7-day period. These findings suggest that argon plasma treatment enhances protein retention capability by promoting stronger binding affinity and more effective physical adsorption of rhSLPI within the micro/nano-architecture compared to the SBTi surface [27].

The cytotoxicity of the surface-modified Ti was determined on human osteoblast cells. The result demonstrated that after SLA modification, there was no toxicity to the cells, including the combined SLA groups (SBPTi, SBTi-rhSLPI, and SBPTi-rhSLPI). Based on the findings, SLA modification, as well as the combination of SLA with plasma treatment and rhSLPI coating, might be safe for further investigation and potential clinical application.

Then the adhesion assay demonstrated that surface modification of Ti, including SLA treatment, plasma treatment, and rhSLPI coating, significantly influenced osteoblast adhesion and morphology at the early stage of cell-material interaction. MTT assay results revealed that the modified Ti groups exhibited significantly greater osteoblast adhesion compared to the unmodified Ti control, suggesting that the characteristic of the surface plays a crucial role in enhancing cell adhesion. Evidence from a previous study indicates that increased hydrophilicity and roughness of the Ti surface can enhance osteoblast adhesion by increasing interactions with adhesion molecules, thereby promoting cell attachment to the implant surface [29]. Morphological observation using FE-SEM further revealed these findings. The human osteoblasts cultured on Ti exhibited a rounded morphology with minimal cytoplasmic spreading, which is characteristic of poor adhesion. In contrast, cells on SBTi surfaces demonstrated initial cytoplasmic extensions, indicating an early stage of attachment and spreading, which is consistent with the previous report that the surface roughness of implant materials has been shown to enhance osteoblast differentiation, as the microstructure generated by SLA modification resembles the extracellular matrix (ECM), which further stimulates osteoblast attachment and spreading [30]. The SLA modification generates ECM-like micro- and nanostructures that promotes the binding of transmembrane integrin receptors, which stimulates intracellular signaling cascades through the focal adhesion kinase (FAK) and paxillin pathways. The activation of this FAK/paxillin pathway leads to cytoskeletal reorganization and cytoplasmic extensions [30,31]. Moreover, the morphology of the cells on the combination of the SLA technique with the plasma treatment (SBPTi) or rhSLPI coating (SBTi-rhSLPI) surfaces exhibited cytoplasmic extensions projecting away from the cell body while maintaining a round central morphology. The previous study suggested that plasma treatment could provide a negative charge on the surface, promoting the adsorption of adhesion molecules, including integrin, fibronectin, and vitronectin, resulting in enhanced cell adhesion and cytoplasmic extension [32]. Additionally, previous studies revealed that rhSLPI could induce human osteoblast cell adhesion and cytoplasmic extension and expansion on the Ti surface through the (FAK) pathway [21,31]. Importantly, in the SBPTi-rhSLPI group, osteoblasts exhibited extensive cytoplasmic extensions and a flattened morphology, which indicated strong adhesion and potential progression toward differentiation [33]. Moreover, the expression of adhesion-related genes demonstrated the positive effect of surface modification on human osteoblast adhesion. In particular, SBPTi-rhSLPI showed significantly higher expression of *Itg*α*1* and *Itg*β*1*, which are the subunits that form the integrin-α1β1 and play a role in integrin-mediated binding to extracellular matrix (ECM) proteins such as collagen and fibronectin, resulting in strengthened cell–implant interactions [34]. The upregulation of these genes suggests that the combination of SLA, plasma treatment, and rhSLPI provides an enhanced effect in promoting integrin signaling. Moreover, Itgα2 expression, which is the collagen-binding integrin, was significantly higher in SBPTi and SBPTi-rhSLPI compared to Ti, with SBPTi exhibiting markedly greater levels than SBTi and SBTi-rhSLPI [35]. Additionally, *Itg*α*2* expression is influenced by implant surface characteristics, including surface roughness and surface energy, consistent with recent findings that only the plasma-treated groups demonstrated significantly elevated expression levels [36]. The *Itg*α*5* expression was upregulated in SBTi-rhSLPI and SBPTi-rhSLPI, which demonstrated the highest expression among all groups. The *Itg*α*5* forms integrin-α5β1, which directly attaches to the ECM and is involved in initial osteoblast cell adhesion that may result in the highest cytoplasmic spreading [37]. Moreover, the upregulation of *Itg*α*5* expression was higher in the rhSLPI-coated groups, suggesting that rhSLPI coating may serve as a contributing factor in stimulating *Itgα5* expression. This finding is consistent with previous studies reporting that rhSLPI coating on titanium surfaces enhances *Itgα5* expression [21]. These findings indicate that the combined modification of SLA, plasma treatment, and rhSLPI coating significantly upregulates adhesion-related genes, thereby supporting enhanced osteoblast adhesion and cytoskeletal reorganization at the early stage of cell-material interaction. Thus, the current findings suggest that combining SLA, plasma treatment, and rhSLPI coating may generate an optimal microenvironment for human osteoblast adhesion and cytoplasmic spreading. Thus, modifications may improve the early biological response to titanium implant materials, potentially enhancing osseointegration in further in vivo studies and clinical applications.

However, the proliferation finding revealed that the modified groups exhibited significantly lower cell proliferation compared to the control Ti. Consistent with the previous finding, this suggested that the osteoblasts cultured on the nano- and micro-scale roughness exhibited decreased cell proliferation and that the cells might shift forward to the next stage of development [9]. However, it is important to note that in vitro results cannot clearly determine whether the newly formed bone possesses sufficient volume and mechanical strength. Therefore, further in vivo animal studies are required to verify the actual tissue healing and bone formation around the implant.

Nevertheless, the Alizarin Red S staining results demonstrated that all modified groups significantly enhanced mineral deposition compared to the unmodified Ti, indicating that surface modification and rhSLPI coating could support osteogenic differentiation. The SLA modification generated the micro- and nano-scale roughness that mimics the ECM, which facilitated osteoblast differentiation [30]. According to a previous study, hydrophilic SLA titanium not only showed higher mineralization in 3 weeks but also increased the expression of osteogenic differentiation genes, including ALP, Runx2, OPN, and OCN, compared to non-modified surface titanium [38]. Furthermore, the combination of SLA and plasma treatment or rhSLPI coating exhibited a higher rate of bone differentiation. The higher mineral deposition in SBPTi could be due to the increased surface roughness generated by SLA treatment, while plasma treatment further enhances hydrophilicity, introduces functional groups, and removes surface contaminants, resulting in enhanced osteoblast activity and mineralization [38,39]. Moreover, after being coated with rhSLPI protein (SBTi-rhSLPI), the deposition increased, which is consistent with the previous finding that rhSLPI could promote osteoblast cell differentiation through the focal adhesion kinase/extracellular signal-regulated kinases 1/2 (FAK/ERK 1/2) pathway [37,40]. Lastly, SBPTi-rhSLPI exhibited the highest mineralization, suggesting an enhanced effect of plasma treatment and rhSLPI in promoting osteoblast differentiation and mineral deposition. These findings indicate that the combination of SLA, plasma treatment, and rhSLPI coating enhances human osteoblast adhesion, cytoplasmic spreading, and mineralization, and may represent an optimal condition for orthopedic and dental implant applications.

The antibacterial adhesion property of the modified titanium was evaluated by determining the metabolic activity of the adhered bacteria against Gram-positive and Gram-negative bacteria using the MTT assay. For *S. aureus*, the results showed that all surface modifications (SBTi, SBPTi, SBTi-rhSLPI, and SBPTi-rhSLPI) reduced bacterial adhesion compared to the untreated Ti control, with no significant differences among the modified groups. This finding suggests that the reduction in *S. aureus* adhesion was attributable to the physical and topographical changes in the surface. The SLA modification and plasma treatment induced micro- and nano-roughness and enhanced surface hydrophilicity. These changes reduced the function of microbial surface components recognizing adhesive matrix molecules (MCRAMM), which play a crucial role in the initial stage of Gram-positive bacterial adhesion to the surface [41]. In contrast, bacterial adhesion of Gram-negative *E. coli* was significantly reduced on rhSLPI-coated surfaces (SBTi-rhSLPI and SBPTi-rhSLPI), while the uncoated surfaces showed no significant difference compared to the control. The results suggests that the physical properties and morphology are insufficient to reduce *E. coli* adhesion, since the *E. coli* possesses multiple adhesion mechanisms, such as pili or fimbriae, that might attach to the macro- and nano-rough surface. However, the reduction in bacterial adhesion in rhSLPI-coated groups is consistent with the hypothesized antimicrobial mechanism of the SLPI, which is reported to disrupt the bacterial cell membrane via its strongly positive charge and to bind to the mRNA and DNA to inhibit vital protein synthesis [18]. Based on the bacterial adhesion findings, the combination of physical surface treatment (SLA modification and plasma treatment) with a bioactive molecule (rhSLPI) coating demonstrates significant potential as an effective surface modification technique, representing a promising candidate for further research and clinical application in medical implants.

The current study was performed to investigate the novel combination strategy on sandblasting, large-grit, and alkaline etching (SLA) and argon-nonthermal plasma treatment and secretory leukocyte protease inhibitor (SLPI) coating on the titanium surface to enhance the surface properties and improve bone cell activities. From this current study, SBPTi-rhSLPI might be suitable for further study in in vivo models and further development for real clinical application in orthopedic and dental implantation. Nevertheless, there are several issues that are considered limitations of this current study, including that some physical and chemical characterizations have not been performed, such as direct chemical characterization by X-ray photoelectron spectroscopy (XPS) or Fourier-transform infrared (FTIR) spectroscopy on the modified surface, as well as assessments of long-term (shelf-life) stability of the sample, corrosion resistance, etc. Although SEM findings provided cell morphology and initial spreading, a cytoskeleton assessment or specific migration should be performed to reveal the dynamic cell-material interactions. Moreover, a recent study has not demonstrated the gene expression of bone differentiation. Furthermore, the antibacterial evaluation was evaluated by the MTT assay, which measures metabolic activity of the bacterial activity rather than providing an absolute bacterial count. Additionally, the evaluation of the modified titanium was performed on healthy cells (hFOB 1.19), which provides a standardized and highly reproducible model for initial biomaterial screening. However, due to its rapid proliferation and high developmental capacity, it does not accurately reflect the compromised healing environment and lower metabolic activity of senescent or osteoporotic bone. Consequently, this study might lack the data for clinical translation. Moreover, this study revealed only the effect of modified titanium on osteoblasts, while bone diseases involve both reduced osteoblast activity and increased osteoclast activity. In addition, the primary anti-protease function of rhSLPI was not investigated. Finally, this study was conducted in an in vitro model as a proof-of-concept. Therefore, further in vivo studies are required to confirm the real effects of combining SLA, plasma treatment, and SLPI coating, and to advance its potential clinical application.

## 5. Conclusions

This study is the first to develop a combination strategy involving sandblasting, large-grit, and alkaline etching (SLA), argon non-thermal plasma treatment, and secretory leukocyte protease inhibitor (SLPI) coating. This combined approach significantly enhanced human osteoblast adhesion, spreading, and mineralization, while simultaneously exhibiting antibacterial adhesion efficacy. Therefore, this study serves as a foundational model for further investigations in orthopedic and dental applications.

## Figures and Tables

**Table 1:** Adhesion molecule primers.

Primers	Sequence		Annealing Temperature (°C)
H-GAPDH	F	GCT CTC CAG AAC ATC ATC C	48.00
	R	TGC TTC ACC ACC TTC TTG	
H-ITGα1	F	CAC TCG TAA ATG CCA AGA AAAG	47.00
	R	TAG AAC CCA ACA CAA AGA TGC	
H-ITGα2	F	ACT GTT CAA GGA GGA GAC	46.00
	R	GGT CAA AGG CTT GTT TAG G	
H-ITGα5	F	ATC TGT GTG CCT GAC CTG	50.00
	R	AAG TTC CCT GGG TGT CTG	
H-ITGβ1	F	TCC TCC TCA TTT CAT TCA TC	45.00
	R	ATT ACT CAG ATC CAA CCA C	

## Data Availability

The original contributions presented in the study are included in the article, further inquiries can be directed to the corresponding author.

## Abbreviations

ALP	Alkaline Phosphatase
BIC	Bone-Implant Contact
BrdU	Bromodeoxyuridine
cDNA	Complementary DNA
DMEM	Dulbecco’s Modified Eagle’s Medium
DMSO	Dimethyl Sulfoxide
DNA	Deoxyribonucleic Acid
*E. coli*	*Escherichia coli*
ECM	Extracellular Matrix
ELISA	Enzyme-Linked Immunosorbent Assay
ERK 1/2	Extracellular Signal-Regulated Kinases 1/2
FAK	Focal Adhesion Kinase
FBS	Fetal Bovine Serum
FE-SEM	Field Emission Scanning Electron Microscope
GAPDH	Glyceraldehyde 3-phosphate dehydrogenase
hFOB 1.19	Human Fetal Osteoblast 1.19
HRP	Horseradish Peroxidase
IgG	Immunoglobulin G
IIPP	International Internship Pilot Program
LB	Luria–Bertani broth
LD10	Lethal Dose 10%
MCRAMM	Microbial Surface Components Recognizing Adhesive Matrix Molecules
mRNA	Messenger RNA
MTT	3-(4,5-dimethylthiazol-2-yl)-2,5-diphenyltetrazolium bromide
OCN	Osteocalcin
OPN	Osteopontin
Pa	Average roughness
PBS	Phosphate-Buffered Saline
PCR	Polymerase Chain Reaction
PECVD	Plasma-Enhanced Chemical Vapor Deposition
rhSLPI	Recombinant human Secretory Leukocyte Protease Inhibitor
RNA	Ribonucleic Acid
ROS	Reactive Oxygen Species
RT-qPCR	Quantitative Reverse Transcription Polymerase Chain Reaction
Runx2	Runt-related transcription factor 2
*S. aureus*	*Staphylococcus aureus*
SBPTi	SLA Titanium combined with Plasma treatment
SBPTi-rhSLPI	SLA Titanium combined with Plasma treatment and rhSLPI coating
SBTi	Sandblasted, large-grit, and alkaline-etched Titanium
SBTi-rhSLPI	SLA Titanium combined with rhSLPI protein coating
SLA	Sandblasting, Large-grit Alkaline etching
SLPI	Secretory Leukocyte Protease Inhibitor
Ti	Titanium and its alloys
TMB	Tetramethylbenzidine
*v*/*v*	Volume per volume
*w*/*v*	Weight per volume
